# The Book World of Medicine and Science

**Published:** 1903-08-15

**Authors:** 


### The Book World of Medicine and Science.

Diseases and Injuries of the Eye. By Lawson. Sixth Edition. (Smith, Elder and Co. 1903. Pp. 587. Illustrations 249.)

The previous edition of this work dates as far back as 1885; during the interval considerable progress has been made in ophthalmology, and as a result the present edition is a much enlarged and in every way an improved one. The work is conveniently divided into 27 chapters and an appendix. The first eight chapters are devoted to optics—examination of the eye and the various anomalies of refraction. Fortunately, the optics and their bearing on ophthalmology are not drawn out *ad nauseam*, as is so usual in text-books of this nature. Quite sufficient is contained in the first chapter of nine pages. The treatment of errors of refraction is sound and not too controversial. This is probably the most difficult department of ophthalmology as evidenced by the very varied advice given by different authorities. What is required by the student and practitioner is a definite statement of treatment and not half-a-dozen expert opinions; in this work he obtains the former, and will not be far wrong in following the advice given. It might have been stated with rather more force that in all errors of refraction before the age of 25 years *the* test is retinoscopy under atropine. The degree of error makes but little difference, especially if there is any astigmatism. Even the oculist of very long standing cannot accurately determine the error without the use of a mydriatic. Strabismus, which is so common in hypermetropia, has been relegated to chapter 24. To the beginner, retinoscopy presents many difficulties, and one is the quality of the shadow: there is no doubt that the shadow in any degree of refractive error is much more clearly defined when a plane mirror is used, instead of a concave one. In dealing with the treatment of myopia there is a valuable page on the removal of the lens for high degrees; the dangers of the treatment are lucidly dealt with, but the results of this operation up to date are not set forth. In describing the many diseases and injuries, and in giving their treatment there is a sense throughout that the work is the result of actual experience without undue regard to all the literature on the subject; this is a pleasing feature, though it may not receive the approbation of the theoretical and argumentative. The attention to minutiæ in describing the technique of operations—on which the success of ophthalmic surgery so largely depends—should be invaluable to the young surgeon; a good example is the description given of enucleation of the eye. It has been well said that the skill shown in this operation is a good indication in the young surgeon as to his fitness to undertake those more special operations having for their purpose the improvement of sight, and when one sees the execrable manner in which "blind eyes" are "pulled out" it is unfortunate that such operators should have a valuable eye at their mercy. It is well to see special stress placed on those "apparently" slight injuries in the region of the roof of the orbit, which have frequently been a surprise in their results and cause of a loss of professional reputation. An interesting chapter is the one on affections of the eyes in diseases of the nervous system, and perhaps one is surprised in such a detailed description to miss the bilateral ptosis frequently found in myasthenia gravis, which has the peculiarity of increasing as the day progresses. With regard to drugs, one might have expected in such an up-to-date work more than two lines on that very useful preparation adrenalin, which has come to stay. In plastic operation on the lids it is most useful in preventing the parts from being constantly obscured by the bleeding. There is also no doubt that it clears nebulous corneæ if applied soon after healing has taken place. These are but two instances of its value. The author's incursion into the realms of rhinology cannot be said to be as successful. Of course the oculist only comes in contact with those cases of frontal sinus trouble which produce displacement of the globe and consequent diplopia; these cases are due either to growth in the sinus or mucocele; pus in the sinus (empyema) very rarely produces distention. The rhinologist of to-day looks askance at a case that has been drained for six months, as he frequently obtains a cure in fewer weeks. The treatment consists in obliterating the sinus so as to allow the anterior fleshy wall to come in contact with the posterior; the resulting deformity is not great and can be remedied by subcutaneous paraffin injections. These few discrepancies do not, however, detract from the general value of the book, which is of a high and useful standard. The exceptionally detailed and voluminous index increases the value of the work as one of reference. To advanced students and practitioners it must prove a most valuable treatise.

Diseases of Women. By G. E. Herman, M.B., F.R.C.P. Revised Edition. Pp. 884. Illustrated. Price 25s. (Cassell and Co., Ltd., London, Paris, etc.)

This revised edition of Dr. Herman's book is exactly what it is intended by the author to be—a guide to the student and practitioner in diagnosing and rightly treating the diseases of women. In it the different diseases which affect the organs peculiar to women are presented in the way in which they appear in practice. Not every thing which has been recommended is mentioned; but principles are stated, and that which in applying those principles has been found good is recommended. The book is in fact written by one who has the prime qualification for writing such a book—clinical experience. The author's style is terse and energetic. There is no laboured pathology or distorted reasoning from anatomy to confuse the student; but diagnosis is made easy and treatment simple. Certain subjects of difficulty are treated with admirable sobriety and restraint, and the counsels that practitioners may give their patients are dignified and judicious. The printing, paper, and illustrations leave little to be desired.

Muco-membranous Colitis. By Dr. Froussard. (London: Baillière, Tindall and Cox. 1903. Pp. 15. Price 1s.)

To those interested in that troublesome form of chronic enteritis known as muco-membranous colitis, we can recommend this pamphlet. Although the author contributes nothing material to the little known of the etiology and pathology of the disease, he so carefully reviews the series of symptoms, that a perusal of the article is instructive. He regards the disease as of nervous, rather than of microbic origin, and in the treatment directs particular attention to the general disturbance of the nervous system.

